# Peroxiredoxin 1 promotes invasion and migration by regulating epithelial-to-mesenchymal transition during oral carcinogenesis

**DOI:** 10.18632/oncotarget.9705

**Published:** 2016-05-30

**Authors:** Wenwen Niu, Min Zhang, Hui Chen, Chunxiao Wang, Ni Shi, Xinying Jing, Lihua Ge, Tong Chen, Xiaofei Tang

**Affiliations:** ^1^ Division of Oral Pathology, Beijing Institute of Dental Research, Beijing Stomatological Hospital and School of Stomatology, Capital Medical University, Dongcheng District, Beijing, China; ^2^ Division of Medical Oncology, Department of Internal Medicine, The Arthur G. James Cancer Hospital and Richard J. Solove Research Institute, The Ohio State University, Columbus, Ohio, USA

**Keywords:** peroxiredoxin 1, NFκB, oral squamous cell carcinoma, epithelial-to-mesenchymal transition, nicotine

## Abstract

Tobacco smoking is the major risk factor for oral squamous cell carcinoma (OSCC). Previously, we found that nicotine up-regulates peroxiredoxin 1 (Prx1), an important antioxidant enzyme, and nuclear factor kappa B (NFκB) in OSCC cells. However, the molecular mechanism of Prx1 in oral carcinogenesis remains obscure. To improve our understanding of the functional role of Prx1 during the cascade of tobacco-associated oral carcinogenesis, we characterized Prx1, NFκB, and epithelial-to-mesenchymal transition (EMT) markers including E-cadherin, vimentin and Snail in 30 primary oral tumors (15 from smokers with OSCC and 15 from non-smokers with OSCC) and 10 normal oral mucosa specimens from healthy individuals. The expression levels of Prx1, nuclear NFκB, vimentin and Snail were higher in the tumors from smokers with OSCC than in those from non-smokers with OSCC or the healthy controls. The expression levels of E-cadherin showed an opposite trend. Prx1 silencing suppressed the nicotine-induced EMT, cell invasion and migration in SCC15 cells *in vitro*. Furthermore, Prx1 activated the NFκB pathway in SCC15 cells. Prx1 might therefore play an oncogenic role in tobacco-related OSCC and thus serve as a target for chemopreventive and therapeutic interventions.

## INTRODUCTION

Oral squamous cell carcinoma (OSCC) is the sixth most common cancer and represents a huge health burden worldwide [1]. The overall survival rate of patients with OSCC is low because of late diagnoses, low therapeutic response rates, and high recurrence [2]. Tobacco smoking is the major risk factor for OSCC and is associated with tumor development, invasion, and metastasis, which are responsible for the high recurrence and poor prognosis [3, 4].

Increasing evidence suggests that epithelial-to-mesenchymal transition (EMT) contributes to the tumor metastatic cascade. EMT involves a series of morphological alterations (losses of cell-cell junctions and cell polarity) and molecular changes including the down-regulation of epithelial cell markers (e.g., E-cadherin) and the up-regulation of mesenchymal adhesion and cytoskeletal proteins (e.g., vimentin) and transcription factors (e.g., Snail) [5, 6]. Enhanced EMT is associated with poor overall and metastasis-free survival in OSCC [7]. Exposure to cigarette-smoke extract can promote the EMT process. The long-term use of nicotine can enhance the expression of Snail, an important EMT regulator, in OSCC cells [8, 9].

Previously, we found that nicotine up-regulates peroxiredoxin 1 (Prx1). Prx1 overexpression was associated with poor prognosis and tumor growth in a xenograft model [10–12]. Prx1 is a major 2-Cys member of the peroxiredoxin family. Aberrant Prx1 expression is reported in numerous cancers including OSCC and cancers of the breast, thyroid, lung, bladder, and prostate [13–18]. The primary biochemical function of Prx1 appears to be peroxide detoxification and reactive oxygen-species scavenging [19]. Prx1 also acts as a molecular chaperone to regulate cell proliferation, differentiation, and apoptosis under stressful conditions [20, 21]. In addition, Prx1 can increase microvascular invasion and tumor-node metastasis. Prx1 stimulates endothelial cell proliferation, migration, and differentiation depending on Toll-like receptor 4 and vascular endothelial growth factors [22, 23]. Studies suggest that Prx1 modulates transforming growth factor-beta 1-induced EMT through its peroxidase activity [24]. Our former study verified that Prx1 regulates the translocation and DNA-binding activity of nuclear factor kappa B (NFκB) in oral cancer cells. NFκB signaling plays an important role in the EMT process by regulating the EMT-related transcriptional factor ZEB1, Snail, and other proteins [25, 26]. Therefore, we hypothesize that nicotine induces EMT in oral cancer by activating the Prx1 and NFκB cascade.

The objective of this study was to improve our understanding of the functional role of Prx1 during nicotine-associated invasion and migration in OSCC. We first compared Prx1 expression between smokers with OSCC and non-smokers with OSCC. We then examined the effect of tobacco smoke on EMT in patients with OSCC. Next, we knocked down Prx1 in an OSCC cell line and determined the effect of the knockdown on oral squamous-cell invasion and migration and the EMT process. Finally, we investigated the effect of Prx1 on the NFκB activation. Our results demonstrate that Prx1 might serve as a novel biomarker and play a critical role in tobacco-related OSCC.

## RESULTS

### Prx1 is overexpressed in human OSCC tissues

**Figure 1:**
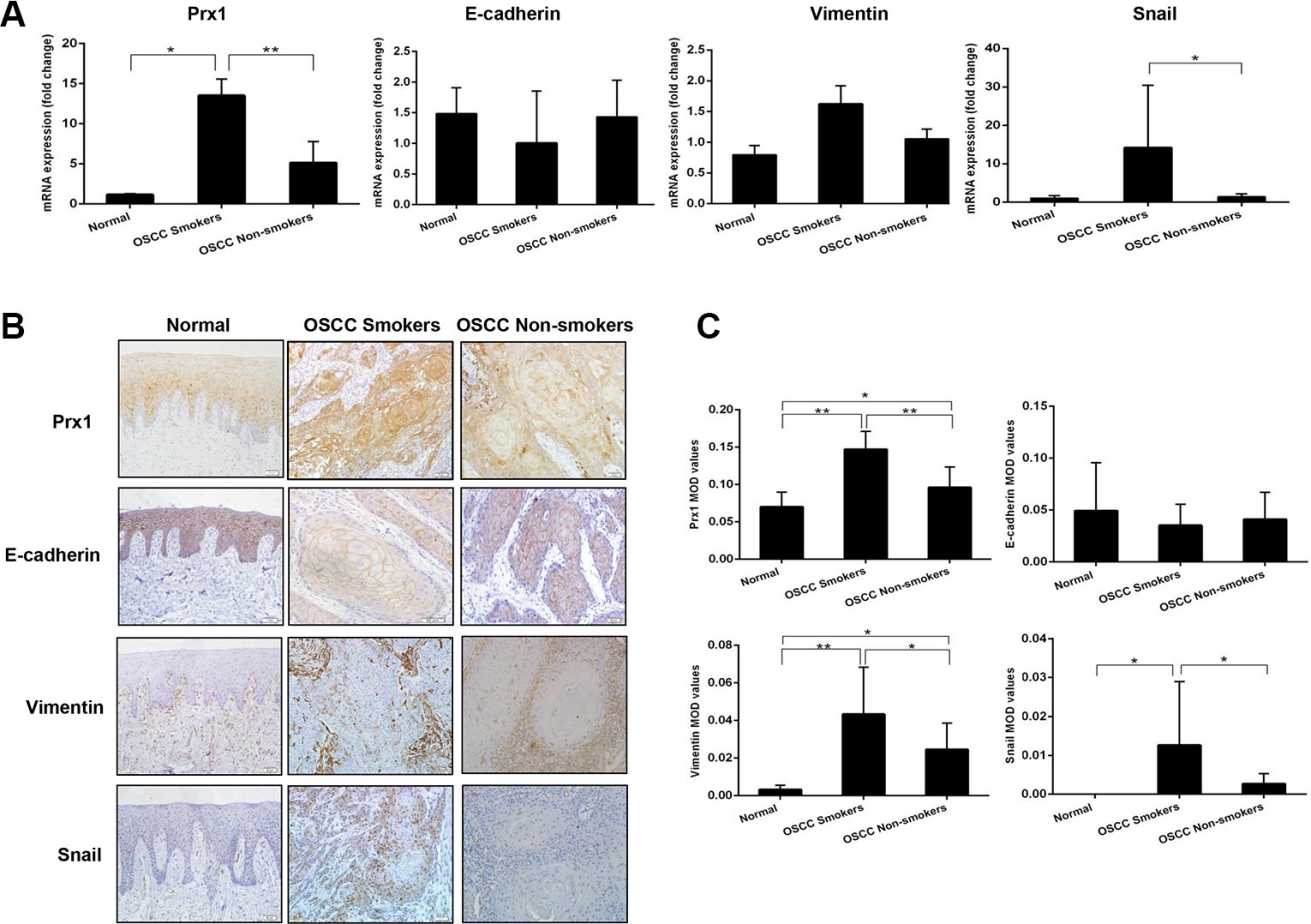
Expression of Prx1 and EMT markers in human OSCC tissues (**A**) mRNA expression of Prx1, E-cadherin, vimentin and Snail in human oral mucosa specimens detected by real-time PCR; (**B**) representative immunohistochemistry images of Prx1, E-cadherin, vimentin and Snail in tumors and normal tissues (200× magnification); and (**C**) immunohistochemistry scores analyzed by mean optical density (MOD). The values are expressed as the mean ± SE. *P < 0.05; **P < 0.01.

We detected Prx1 expression in oral-mucosa specimens obtained from 15 smokers with OSCC, 15 non-smokers with OSCC, and 10 healthy individuals. The Prx1 mRNA expression level was significantly elevated in the smokers and non-smokers with OSCC compared with that in the healthy individuals (Figure 1A). Immunohistochemistry analysis revealed comparable levels of Prx1 protein overexpression among the same tissues (Figure 1B and 1C). The Prx1 expression was lowest in the healthy individuals, higher in the non-smokers with OSCC, and highest in the smokers with OSCC.

### The EMT markers E-cadherin, vimentin and Snail are altered in human OSCC tissues

We characterized the expression of E-cadherin, vimentin and Snail in oral mucosa specimens. The smokers and non-smokers with OSCC had lower expression levels of E-cadherin mRNA and higher expression levels of vimentin and Snail mRNAs compared with the healthy individuals (Figure 1A). The immunoreactivities for vimentin and Snail were lowest in the healthy individuals, higher in the non-smokers with OSCC, and highest in the smokers with OSCC, whereas that for E-cadherin displayed the opposite trend (Figure 1B and 1C).

### Nicotine increases Prx1, the EMT process, cell invasion, and migration *in vitro*

**Figure 2:**
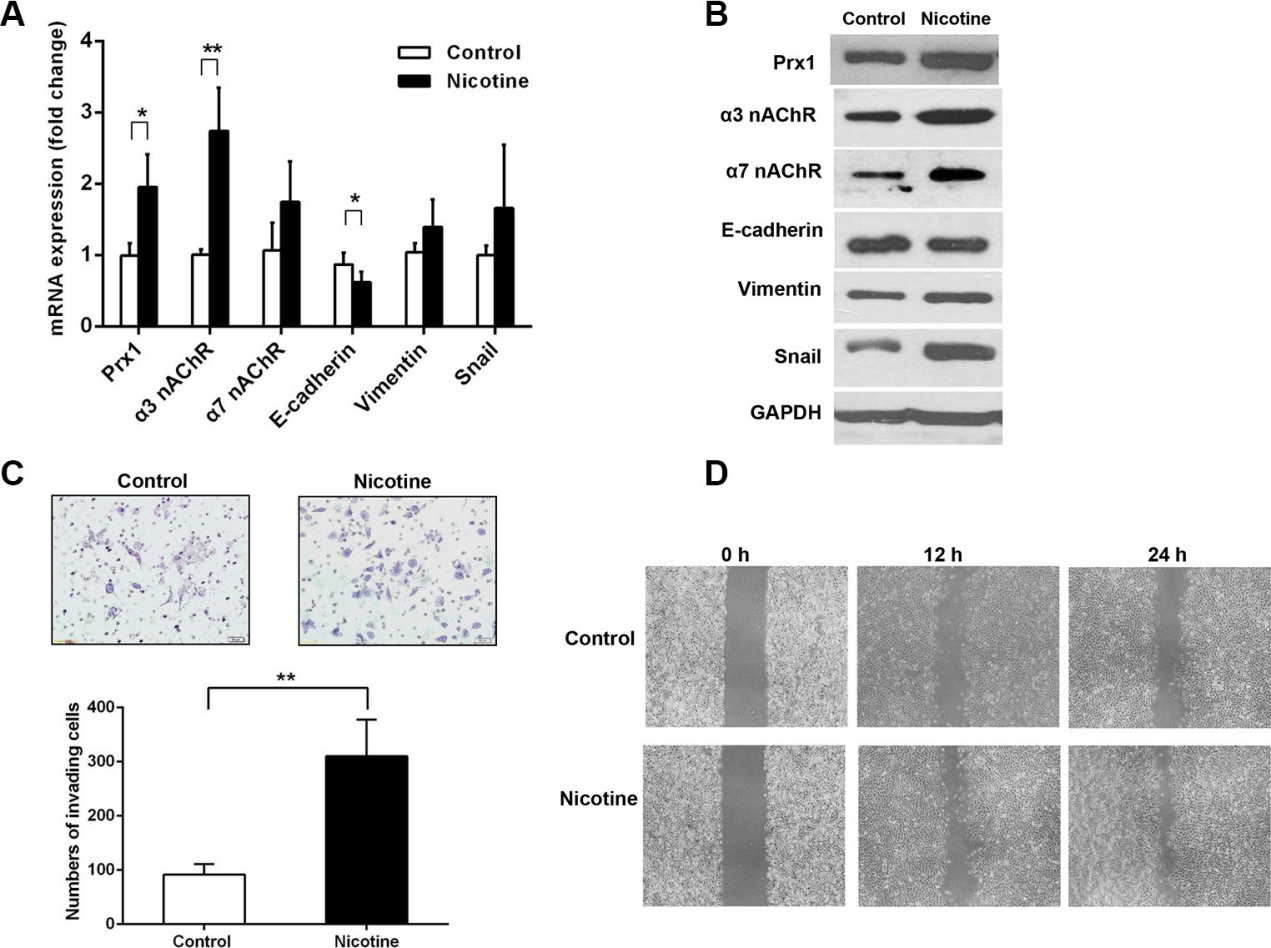
Nicotine up-regulates Prx1, α3 nAChR and α7 nAChR, enhances EMT, and promotes invasion and migration in SCC15 cells (**A**) mRNA expression of Prx1, α3 nAChR, α7 nAChR, E-cadherin, vimentin and Snail in control and nicotine-treated SCC15 cells; (**B**) representative blots from one of three separate experiments for the protein expression of Prx1, α3 nAChR, α7 nAChR, E-cadherin, vimentin and Snail in control and nicotine-treated SCC15 cells; (**C**) images of invading control and nicotine-treated SCC15 cells detected by Matrigel invasion assay (upper panel) and statistical analysis (lower panel); and (**D**) wound healing assay to examine the effect of nicotine on SCC15 cell mobility. The values are expressed as the mean ± SE. *P < 0.05; **P < 0.01.

To determine whether nicotine can modulate Prx1, nicotinic acetylcholine receptor (nAChR), and the EMT process, we assessed the mRNA and protein expression of Prx1, α3 nAChR, α7 nAChR, and EMT markers (E-cadherin, vimentin and Snail) in SCC15 cells exposed to nicotine. Nicotine increased the mRNA and protein expression of Prx1, α3 nAChR, α7 nAChR, vimentin and Snail, and reduced the mRNA and protein expression of E-cadherin compared with that in control cells (Figure 2A and 2B).

We performed a Matrigel invasion assay to evaluate squamous-cell invasion after nicotine exposure. More SCC15 cells penetrated through the filters after the nicotine treatment compared with control cells (Figure 2C). We performed a wound-healing assay to determine whether nicotine can promote SCC15 cell mobility. Compared with those of control cells, the healing and migration rates of nicotine-treated SCC15 cells increased after 12 and 24 h, respectively (Figure 2D).

### Prx1 knockdown inhibits nicotine-induced EMT, cell invasion, and migration *in vitro*

**Figure 3:**
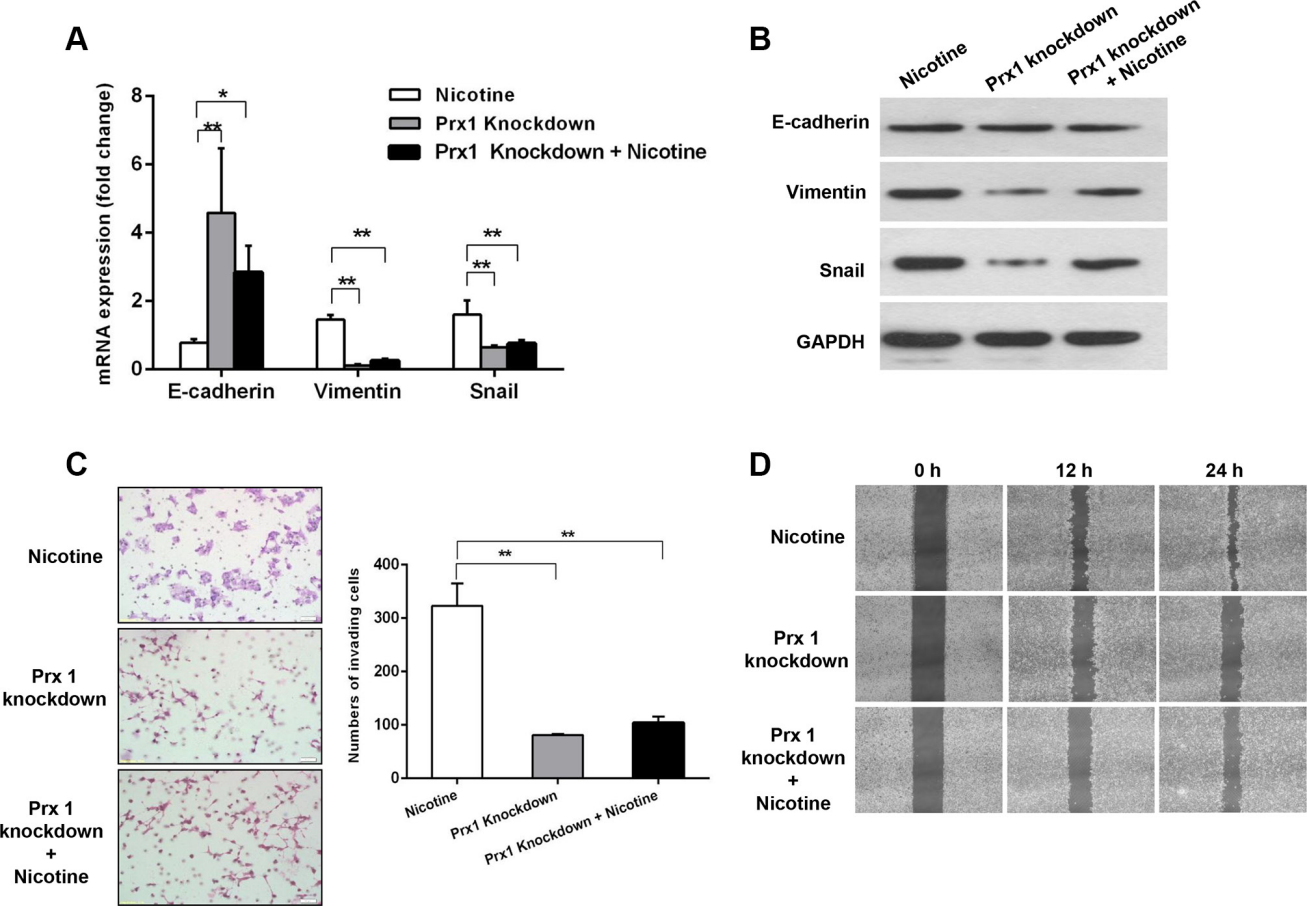
Effects of Prx1 knockdown on nicotine-induced EMT, invasion, and migration in SCC15 cells mRNA (**A**) and protein (**B**) expression of E-cadherin, vimentin and Snail in nicotine-treated control cells, Prx1-knockdown cells, and Prx1-knockdown + nicotine cells. (**C**) images of the invading cells detected by Matrigel invasion assay (right panel) and statistical analysis (left panel); and (**D**) wound healing assay to examine the effect of Prx1 knockdown on SCC15 cells treated with nicotine. The values are expressed as the mean ± SE. *P < 0.05; **P < 0.01.

Prx1 knockdown enhanced the nicotine-induced reduction of E-cadherin expression (*P* < 0.05) and decreased the nicotine-induced overexpression of vimentin and Snail (*P* < 0.01; Figure 3A and 3B). Moreover, Prx1 knockdown reduced the rates of nicotine-induced cell invasion and migration (Figure 3C and 3D).

### Prx1 activates NFkB signaling and promotes EMT, cell invasion, and migration *in vitro*

**Figure 4:**
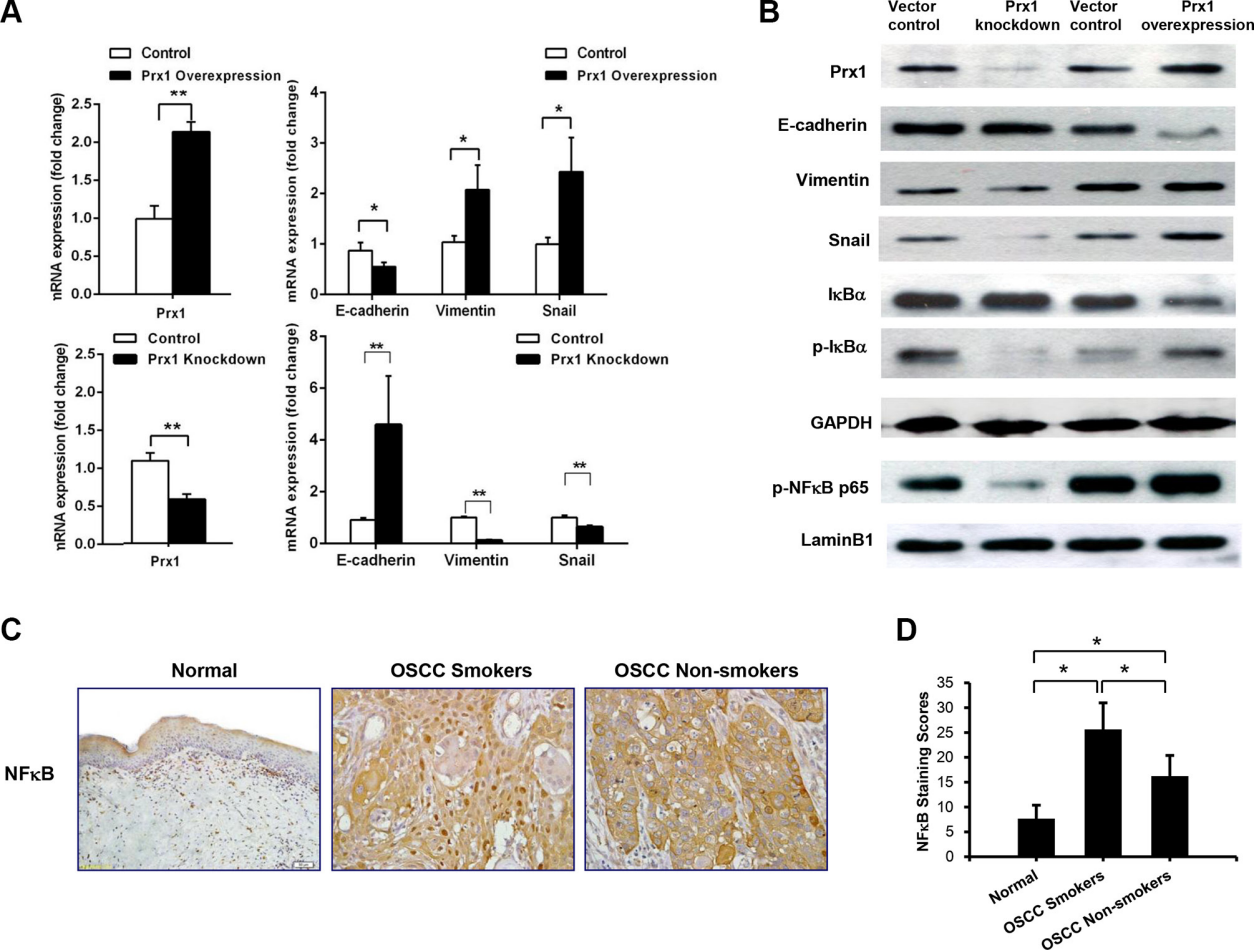
Prx1 activates NFκB signaling and promotes EMT (**A**) mRNA expression of Prx1, E-cadherin, vimentin and Snail in control cells, Prx1-overexpressed cells, and Prx1-knockdown cells detected by real-time PCR; (**B**) western blots for the expression of Prx1, E-cadherin, vimentin, Snail, IκBα, p-IκBα, p-NFκB p65, LaminB1, and GAPDH in control cells, Prx1-overexpressed cells, and Prx1-knockdown cells; (**C**) representative immunohistochemistry images of NFκB in tumors and normal tissues (200× magnification); and (**D**) NFκB staining scores. The values are expressed as the mean ± SE. *P < 0.05; **P < 0.01.

To investigate whether Prx1 modulates EMT in OSCC, we monitored E-cadherin, vimentin and Snail in SCC15 cells that were transfected with Prx1 overexpression plasmid or Prx1 shRNA. We first confirmed the alteration of Prx1 after transfection by real-time PCR and western blot (Figure 4A and 4B). Transfection with the Prx1 overexpression plasmid decreased the expression of E-cadherin and increased the expression of vimentin and Snail. In contrast, transfection with the Prx1 shRNA increased the expression of E-cadherin and decreased the expression of vimentin and Snail (Figure 4A and 4B).

To further explore the molecular mechanisms responsible for Prx1-mediated EMT, we examined the activation of NFκB in SCC15 cells with altered Prx1 expression. Nuclear p-NFκB p65 and p-IκBα were significantly up-regulated in Prx1-overexpressed cells. Prx1 knockdown dramatically decreased expression levels of p-NFκB p65 and p-IκBα (Figure 4B). We also evaluated NFκB in human OSCC tissues. IHC staining indicated that the nuclear NFκB expression in oral mucosa was lowest in the healthy control tissues, higher in the non-smokers with OSCC, and highest in the smokers with OSCC, which is similar to the Prx1 expression pattern (Figure 4C and 4D).

**Figure 5:**
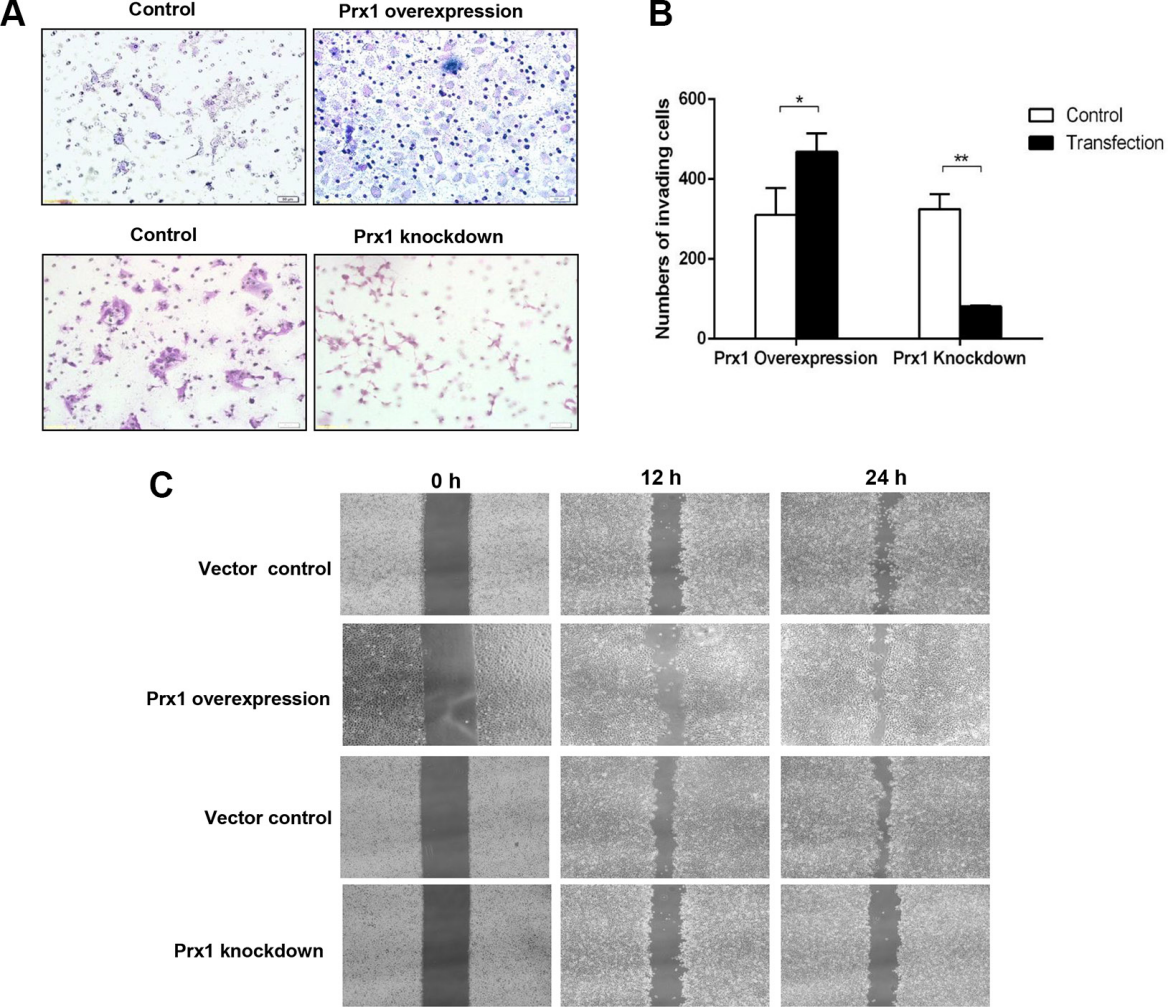
Oral squamous cell invasion and migration are altered by Prx1 *in vitro* (**A**) images of invading SCC15 cells; (**B**) statistical analysis; and (**C**) wound healing assay to examine the effects of Prx1 overexpression and knockdown on SCC15 cell mobility. The values are expressed as the mean ± SE. *P < 0.05; **P < 0.01.

We conducted further Matrigel invasion and wound-healing assays using SCC15 cells with altered Prx1 expression. More Prx1-overexpressed cells than control cells penetrated through the filters after 24 h. Prx1 knockdown decreased the number of invading cells (Figure 5A and 5B). Similarly, the healing and migration rates of SCC15 cells were increased by Prx1 overexpression and decreased by Prx1 silencing compared with those of control cells (Figure 5C).

## DISCUSSION

Nicotine can induce cell proliferation, invasion, and metastasis in several cancers [27, 28]. Nicotine-induced Prx1 overexpression correlates significantly with OSCC carcinogenesis [12, 29], and further investigation of the functional role of Prx1 could provide a novel biomarker for OSCC prevention and therapy.

Nicotine exhibits its pathobiological effects by displacing the local cytotransmitter acetylcholine from the nAChR expressed on the surface of oral epithelial cells [30, 31]. The nAChR subunit family is composed of 17 members: α1–α10, β1–β4, δ, γ, and *ε*. The α3, α5, α7, α9, β2, and β4 subunits are found in human oral epithelial cells. α7 nAChR is the main subtype receptor for tobacco products, and α3 nAChR is another key receptor in oral epithelial cells [31, 32]. The inhibition of α7 nAChR might provide a feasible approach for preventing the progression of head and neck cancer [33]. Previously, we showed that the oral cancer tissues of smokers had higher expression of α3 and α7 nAChR compared with that of non-smokers [34]. Together with those results, the overexpression of α3 and α7 nAChR in SCC15 cells exposed to nicotine suggests that nicotine might up-regulate Prx1 through α3 and α7 nAChR activation and thus serves as a tumor promoter in OSCC.

Metastasis is essentially dependent on the EMT process [5, 6]. During EMT, oral epithelial cells lose their specific phenotypes and develop features of mesenchymal cells. The tight-junction and adherens-junction proteins (e.g., E-cadherin) are down-regulated, while mesenchymal cell-specific proteins (e.g., vimentin) are up-regulated. EMT is enhanced by transcriptional repressors (e.g., Snail) that directly regulate genes involved in cellular adhesion, migration, and invasion [35, 36]. The differential expression levels of Prx1, E-cadherin, vimentin and Snail between smokers and non-smokers with OSCC, and the effects of Prx1 silencing or Prx1 overexpression on the ability of nicotine to enhance EMT, invasion, and migration in SCC15 cells suggest that Prx1 is a key modulator in the nicotine-induced EMT process.

**Figure 6:**
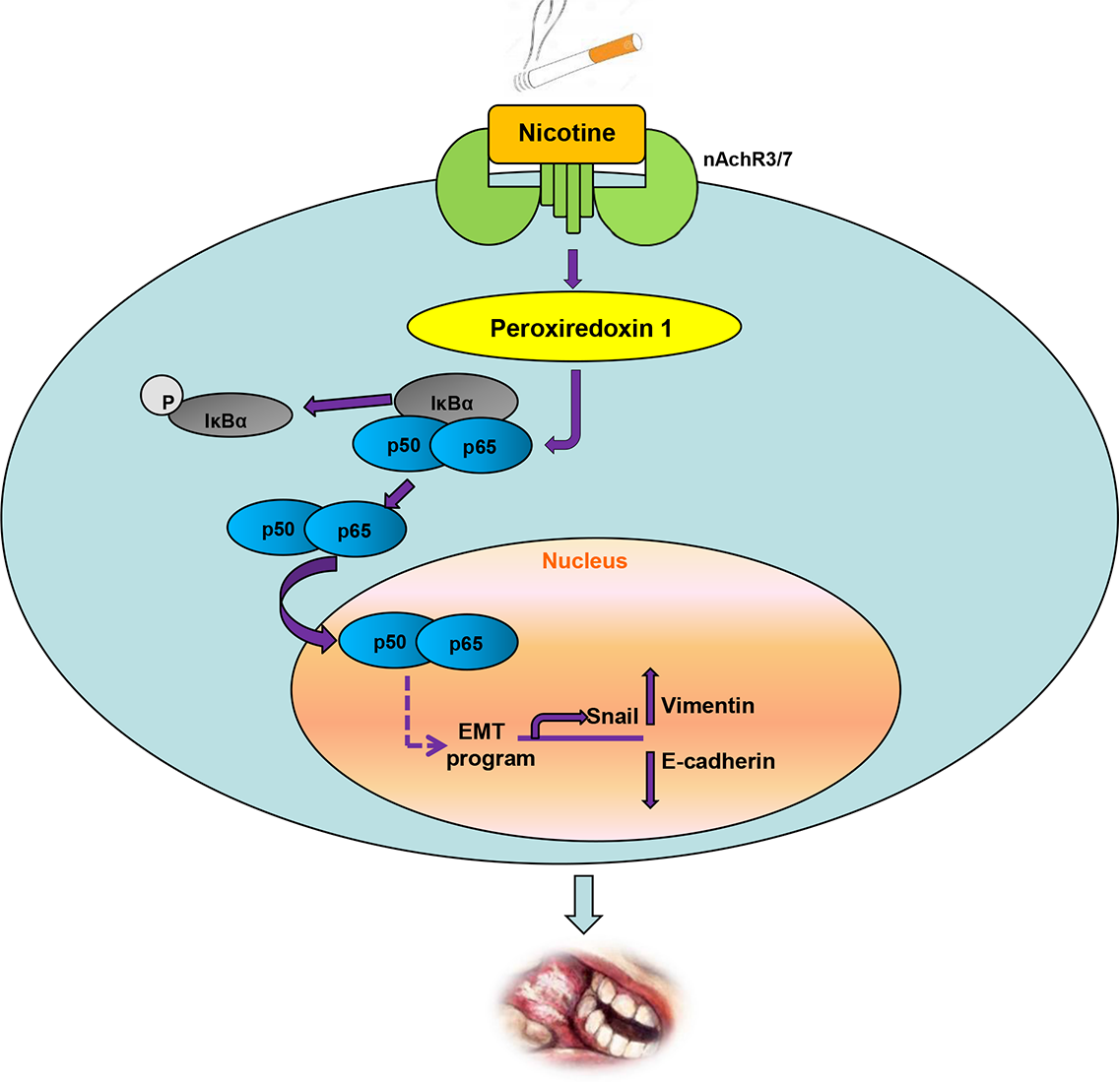
Schematic representation of up-regulation of Prx1 and activation of NFκB by nicotine to promote EMT in OSCC

NFκB is an important transcription factor involved in oncogenic pathways including inflammation, cell differentiation, tumorigenesis, and EMT [37, 38]. For example, NF-κB activation was needed for IL-17-induced EMT, cell migration, and invasion in lung cancer [25]. Zipper-interacting protein kinase increased the expression of β- catenin, Snail and Slug; decreased the expression of E-cadherin; and promoted EMT and metastasis by activating the NFκB pathway [26]. The relatively high expression levels of Prx1 and NFκB in the oral mucosa of smokers with OSCC and the effects of Prx1 alteration on p-IκBα and NFκB nuclear translocation indicate that with nicotine induction, Prx1 is overexpressed and activates NFκB. The NFκB translocates into the nucleus and regulates transcription factors such as Snail to alter the expression of the EMT marker genes E-cadherin and vimentin, resulting in increased invasiveness and migration. We elucidated this mechanism in Figure 6.

In conclusion, Prx1 and NFκB were overexpressed in human oral cancer cells. The expression of Prx1 and NFκB in smokers with OSCC was significantly higher than that in non-smokers with OSCC. Prx1 knockdown suppressed nicotine-induced cell invasion and migration by inhibiting the EMT process *in vitro*. Prx1 promotes the EMT process by activating NFκB activity. Our results suggest that Prx1 plays an oncogenic role in nicotine-enhanced EMT in OSCC. This information could be used to help develop preventive/therapeutic agents that target Prx1 in nicotine-associated diseases including OSCC.

## MATERIALS AND METHODS

### Patients and tissue specimens

The Human Research Ethics Committee of the Capital Medical University School of Stomatology approved all protocols involving human subjects. Informed consent was obtained for all investigations involving human subjects. Primary tumors and normal oral mucosa samples were obtained from 30 patients with OSCC (15 OSCC smokers and 15 non-smokers) and 10 healthy individuals who underwent maxillofacial plastic surgery at the hospital of the Capital Medical University School of Stomatology. All OSCC tissues were moderately to well differentiated. The patient characteristics and clinical information are summarized in Table 1. For histopathologic and immunohistochemical analyses, the oral mucosa specimens were collected immediately after surgical removal and fixed with 10% formalin followed by paraffin-embedding. For real-time PCR analysis, fresh specimens were frozen in liquid nitrogen and stored at −80°C until further use.

**Table 1:** Clinical characteristics of the patients with OSCC

	OSCC Smokers	OSCC Non-smokers
**No. of patients**	15	15
**Age**		
Mean	50	60
Range	37–77	49–75
**Gender**		
Female	0	11
Male	15	4
**Grade**		
Moderate	5	8
Moderate-High	8	2
High	2	5
**Lesion Location**		
Mouth floor	3	0
Buccal	3	1
Gingival	4	3
Tongue	4	8
Maxilla	1	3

### Immunohistochemistry

Oral mucosa specimens were fixed in 10% neutral-buffered formalin overnight, embedded in paraffin, and serially sectioned at 4 μm as described previously [12]. Deparaffinized sections were briefly heated for 10 min in a pressure cooker containing 10 mM citrate buffer (pH 6.0) for antigen retrieval and then soaked in 3% H_2_O_2_ in 0.1 M TBS (pH 7.4) for 15 min to quench endogenous peroxidases. The sections were subsequently treated with protein block solution (Boshide, China) for 20 min and then incubated overnight at 4°C with antibody for Prx1, α3 nAChR, α7 nAChR, Snail (abcam, USA), E-cadherin (Cell Signalling Technology, USA), vimentin (Maixin, China), or p-NF-κB p65 (Cell Signalling Technology, USA). Incubation with primary antibody was followed by incubation for 30 min with horseradish peroxidase-linked secondary anti-rabbit GT Vision^™^ polymer (Gene Tech, USA). The sections were developed with diaminobenzidine (Gene Tech, USA) chromogen and then counterstained with hematoxylin, dehydrated, and mounted for Olympus BX61 microscope observation (Olympus, Tokyo, Japan). The mean optical density was determined by the mean value of positive expression counted in five randomly selected fields. The NFκB staining score (range 0-100) was determined and expressed the percentage of cells with positive p-NF-κB p65 expression.

### Quantitative real-time PCR

Total RNA was extracted from oral-mucosa specimens or cells using TRIzol (Invitrogen Life Technologies, USA) according to the manufacturer's instructions. cDNA was synthesized using a High Capacity cDNA Reverse Transcription Kit (Applied Biosystems, USA). The expression of Prx1, α3nAChR, α7nAChR, E-cadherin, vimentin, Snail, and GAPDH was determined using SYBR Green. The primers for the genes of interest were: Prx1 (Forward 5′-gggtattcttcggcagatca-3′, Reverse 5′-tccccatgtttgtcagtgaa-3′), α3nAChR (Forward 5′-GGACGGGATGTGTGGTTACT-3′, Reverse 5′-TGG CTTCTTTGATTTCTGGTG-3′), α7nAChR (Forward 5′-AAACTCACAGATGGGCAAGG-3′, Reverse 5′-CCGT AAGCAACACGACTGAC-3′), E-cadherin (Forward 5′-TTGCTACTGGAACAGGGACA-3′, Reverse 5′-GTAT TGGGAGGAAGGTCTGC-3′), vimentin (Forward 5′-GAA GAGAACTTTGCCGTTGA-3′, Reverse 5′-CGAAGGTGA CGAGCCATT-3′), Snail (Forward 5′-TTACCTTCCAG CAGCCCTAC-3′, Reverse 5′-GACAGAGTCCCAGATG AGCA-3′), and GAPDH (Forward 5′-AGGTCGGTGTGA ACGGATTTG-3′, Reverse 5′-TGTAGACCATGTAGT TGAGGTCA-3′). Each sample was analyzed in triplicate on an ABI PRISM 7900 (Applied Biosystems).

### Cells and cell treatment

The human OSCC cell line SCC15 was purchased from the American Type Culture Collection. SCC15 cells were cultured in a 1:1 mixture of Dulbecco's modified Eagle medium and Nutrient Mixture F-12 medium + 15% fetal bovine serum (FBS) (Gibco, USA) in a 37°C and 5% CO_2_ environment. The transfected and non-transfected SCC15 cells (1 × 10^6^) were treated with 1 μmol/L nicotine for 7 days.

### Plasmids and cell transfection

To overexpress Prx1, SCC15 cells were transfected with pEZ-M02-PRX1 (GeneCopoeia, USA) or control plasmid (GeneCopoeia, USA) with Lipofectamine™2000 (Invitrogen, USA) to 50% confluence on a 6-well plate. After transfection for 48 h, stably transfected cells were selected using G418 (200 μg/ml) for 10 days. To knock down Prx1, SCC15 cells were transfected with Prx1 shRNA Plasmid (Santa Cruz Biotechnology) using Lipofectamine™2000 (Invitrogen, USA). The shRNA Plasmid-A (Santa Cruz Biotechnology) was used as a control. The efficiency of Prx1 knockdown was determined by RT-PCR and western blot analysis.

### Western blot analysis

Proteins were extracted from cells using immunoprecipitation assay buffer (50 mM Tris-Cl, 1% NP40, 150 mM NaCl, 1 mM EDTA, 1 M phenylmethylsulfonyl fluoride, 10 μg each of aprotinin and leupeptin, and 1 mM Na3VO4). The protein concentration was determined using the Lowry method. Equal amounts of protein were separated on 12% SDS-PAGE gels and blotted onto nitrocellulose membranes. The blots were incubated with primary antibody for Prx1, α3 nAChR, α7 nAChR, Snail (Abcam, USA), E-cadherin (Cell Signalling Technology, USA), vimentin (Bioss, China), IκBα, p-IκBα, p-NFκB p65 (Cell Signalling Technology, USA), or GAPDH (Sigma, USA). The immunoreactive bands were detected with horseradish peroxidase-conjugated secondary antibodies and enhanced chemiluminescence reagents (Amersham Biosciences, USA).

### Matrigel invasion assay

Cell-invasion assays were performed in triplicate using 24-well Transwells (8-mm pore size; Corning, USA) coated with Matrigel (1 mg/ml; BD, USA). SCC15 cells (10^5^ cells/well) were seeded in the upper chambers in culture media containing 0.2% FBS. The lower chambers were filled with 500 μl 10% FBS medium to induce cell migration. Invasion assays were carried out for 24 h for Prx1-overexpressed cells and 48 h for Prx1-knockdown cells. Cells inside the chambers were removed with a cotton swab. The invading cells were stained with Haematoxylin (Salarbio, China) and examined by microscopy (Olympus IX71). Six randomly selected fields were photographed at 200× magnification, and the invading cells were counted. The average number of invading cells in each field represented the invasive ability.

### Wound healing assay

After transfection with Prx1 overexpression plasmid or PrX1 shRNA, OSCC cells were wounded by a 10 μl sterile pipette tip and washed in PBS to remove cellular debris. The cells were then cultured for 24 h. The cells were photographed at 0, 12, and 24 h after wounding.

### Statistical analysis

The mRNA and/or protein expression levels of Prx1, α3 nAChR, α7 nAChR, E-cadherin, vimentin, Snail, IκBα and NFκB, and the data from the invasion and wound healing assays were compared by Student's *t*-tests. All statistical analysis was carried out using SPSS Software for Windows 17.0. Differences were considered statistically significant at *P* < 0.05. All *P* values were two-sided.
